# Vertebral and pelvic echinococcosis in northwestern China

**DOI:** 10.1007/s00264-023-05731-y

**Published:** 2023-02-24

**Authors:** Laihong Yang, Pahati Tuxunjiang, Wenya Liu, Hui Guo

**Affiliations:** 1 Medical Imaging Center, Xinjiang Medical University Affiliated First Hospital, Urumqi, 830054 People’s Republic of China; 2 State Key Laboratory of Pathogenesis, Prevention and Treatment of High Incidence Diseases in Central Asia, Xinjiang Medical University, Urumqi, People’s Republic of China

**Keywords:** Echinococcosis, Human, Vertebra, Pelvis, Bone

## Abstract

**Purpose:**

Echinococcosis remains a major economic and severe public health problem in endemic areas. Bone echinococcosis is rare, and the vertebra and pelvis are the most common sites of echinococcosis involving the skeletal. Because of the clinical severe symptoms and high recurrence rate, it brings excellent trouble to patients.

**Methods:**

This study retrospectively analyzed the clinical manifestations, laboratory tests, radiological findings, and treatment of 44 patients with vertebral and pelvic echinococcosis during a period of 16 years (2005–2020).

**Results:**

The mean age was 43 years (25 males, 19 females; 19–68 years). The most common symptom was pain, followed by numbness, weakness, activity limitation, and progressive paraparesis. Enzyme-linked immunosorbent assay test (ELISA) results were positive in 18 cases (75%). There are 24 cases of hydatid infection of the spine, 14 hydatid infection of the pelvis, and six hydatid infection of both vertebra and pelvis. The site of infection was 13 (29.5%) thoracic, five (11.4%) lumbar, four (9.1%) lumbosacral, seven (15.9%) sacral, 19 (43.2%) ilium, seven (15.9%) hip, six (13.6%) ischium, five (11.4%) pubis, and two (4.5%) femur, respectively. The imaging findings were cystic dilatancy, septal, and irregular bone destruction. MRI has a special value in showing the relationship between the surrounding tissues and organs of cystic bone echinococcosis. All patients were followed up for at least one year. The mean follow-up time was 3.6 years.

**Conclusions:**

Even in epidemic areas, the incidence of bone echinococcosis is relatively rare. However, when encountering the vertebral and pelvic destruction, consider bone echinococcosis’s possibility, especially for the herdsmen in endemic regions.

## Introduction

Hydatidosis is a complex zoonotic parasitic disease caused by *Echinococcus granulosus*. It is prevalent worldwide and a very significant problem in northwest China, where there are more pastoral areas. It can appear in any part of the body, but most hydatidosis occur in the liver (50–77%), followed by the lungs (8.5–43%) [1]. The incidence of bone echinococcosis is estimated at 0.5% to 4% of all reported cases. About 50% of hydatids involve the spinal and pelvic bones. There are no clinical features in the early stage, but with the progression of the disease, it can cause deformation of the spine and pelvis, paralysis of the lower limbs, and so on. When the parasite invades the spine and pelvis, it grows slowly in the bone, spreads along the trabecular cancellous bone, and reaches the bone through the medullary canal. The result is infiltration of vesicles of different sizes without an outer membrane [4]. Patients are usually not easy to detect early. When they come to the hospital, they often find extensive lesions. In addition, because hydatids are rare, doctors often diagnose other diseases. Secondly, this disease is difficult to eradicate and may not be curable, so more attention must be paid to it. Clinical history, radiology, and laboratory tests play an important role in early diagnosis. However, due to the lack of specificity of early clinical symptoms, clinicians often have low awareness of the disease, which often leads to initial misdiagnosis. The aim of this study was to retrospectively analyze the clinical data of 44 cases of echinococcosis of the spine and pelvis diagnosed in our hospital from January 2005 to December 2020, including clinical signs, laboratory, and imaging manifestations and treatment.

## Materials and methods

### Study design

This was a retrospective study. Patients who were pathologically confirmed for the vertebral and pelvic echinococcosis between January 2005 and December 2020 were initially considered eligible for our research. The hospital is the third-grade hospital in Urumqi city, a core district located in the northwest of China. Ethical approval for the study was obtained from the ethical review committee for the hospital, without specific consent from patients.

**Fig. 1:**
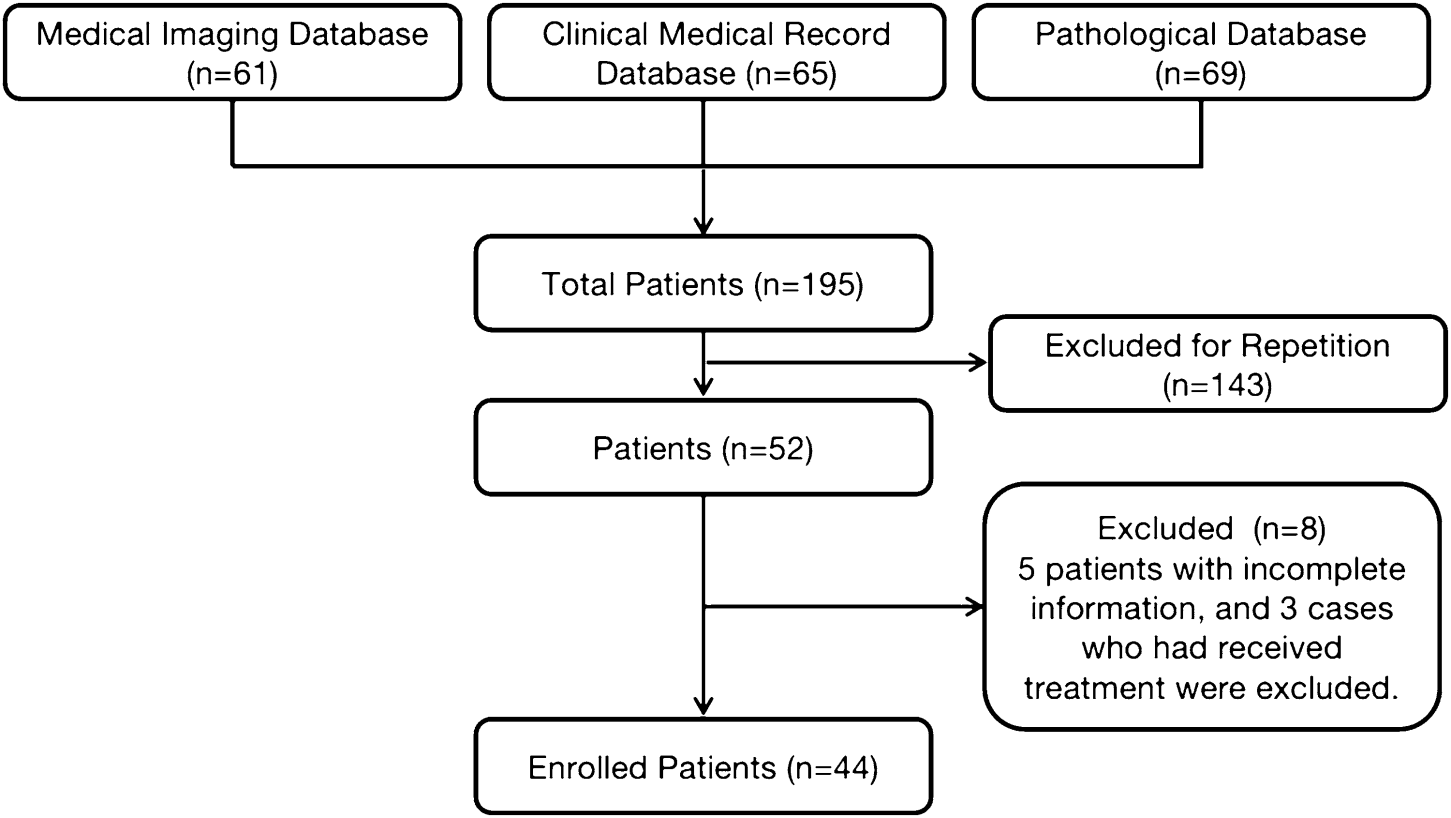
Flowchart of the study population with vertebral and pelvic echinococcosis

Inclusion criteria were as follows: (1) All patients were operated on, with sufficient histopathologic information; (2) All patients did not receive medication, surgery, or radiation therapy at the time of initial enrollment; (3) Information on all patients is complete. A total of 44 patients who met the inclusion criteria were enrolled in our study (Fig. 1).

### Study methodology

The information of 44 patients included gender, age, area of origin, clinical presentation, diagnostic methods, surgical and medical management, and outcome were analyzed using descriptive statistics. Thirty-eight patients performed X-Ray, and 40 patients also underwent computed tomography (CT). Sixteen cases were investigated with contrast-enhanced CT scans. Thirty-five patients underwent magnetic resonance imaging (MRI). Sixteen cases were investigated with contrast-enhanced MR scans. All patients who were followed up by telephone or readmission after discharge were followed up for at least one year.

### Statistical analysis

In this study, the continuous variables were analyzed by average or median, and the categorical variables were analyzed by numerical percentage.

## Results

### Epidemiological and clinical features

The findings in the individual patients were shown in Table 1. Of the 44 patients, 25 (56.8%) cases were male and 19 (43.2%)were female (mean 43 years,age range 19–68 years). most cases were of Han nationality, followed by Kazak, Uygur, Mongolian, Hui, dongxiang and Xibo. 72.7% were rural, and more than half of the patients were farmers. Four patients had a history of close contact with sheep and dogs. Thirty-four cases had a surgical history for hydatid infection, and four patients had a surgical history of hydatid infection in multiple sites,all of whom were treated with albendazole after operation.

**Table 1:** Summary of demographic and clinic characteristics patients (*n* = 44) with the vertebral and pelvic echinococcosis

Case no	Sex/age	Ethnic	Symptoms	Duration of symptoms	Living area	History	ELISA	Diagnosis	Location	Soft tissues	Drug	Surgery	Follow -up	Relapse
1	M/61	Han	Pain	1 mo	R	Lung	−	DR + CT + MRI	T2 − T3 + ribs	Y	Y	Y	1 yr	0
2	M/51	Dongxiang	Pain + numbness	1 mo	R	Lung, liver, and thoracic vertebra	−	DR + CT + MRI	T10 − T11		Y	Y	7 yr	2
3	M/57	Han	Numbness	2 mo	U	Lung		DR	T1 − T3		Y	Y	1 yr	0
4	M/55	Mongols	Pain + weakness	3 mo	R	Thoracic vertebra	−	DR + CT + MRI	T11 − T12 + ribs		Y	Y	5 yr	1
5	M/48	Xibo	Pain	3 d	R	Lung, liver, and thoracic vertebra	+	DR + CT + MRI	T5 − T7 + ribs	Y	Y	Y	2 yr	3
6	M/38	Han	Pain	3 mo	U		−	DR + CT + MRI	T7 − T8 + ribs	Y	Y	Y	2 yr	0
7	M/38	Han	Pain + paraparesis	1 mo	R	Thoracic vertebra	+	DR + CT + MRI	T7 − T11 + ribs	Y	Y	Y	1 yr	2
8	M/31	Kazakh	Pain	3 mo	R	Lung	+	DR + CT + MRI	T6 − T7 + ribs	Y	Y	Y	1 yr	0
9	F/46	Kazakh	Pain + paraparesis	3 mo	U	Lung	+	DR + CT + MRI	T2 − T3 + ribs	Y	Y	Y	1 yr	0
10	M/38	Han	Pain	1 mo	R	Liver	+	DR + CT + MRI	T6 − T8 + T10 − T11	Y	Y	Y	2 yr	0
11	M/61	Han	Pain	3 mo	U	Liver + lung	+	DR + CT + MRI	T3 − T5 + T8 + ribs		Y	Y	2 yr	0
12	F/67	Han	Pain + weakness	2 mo	U	Lung		CT	T5 − T8	Y	Y	Y	22 yr	0
13	F/31	Han	Pain	1 mo	U	Thoracic vertebra		MRI	T6 − T7		Y	N	1 yr	1
14	F/43	Kazakh	Pain	10 yr	R	Lung	+	DR + CT + MRI	L3 − S1 + ilium + pubis + hip	Y	Y	Y	3 yr	0
15	F/28	Kazakh	Pain	2 mo	R			DR + CT + MRI	L2 − L5		Y	Y	1 yr	0
16	F/25	Han	Pain	1 mo	R	Thoracic vertebra		CT	L1 + L3 − L5	Y	Y	Y	7 yr	1
17	F/68	Han	Pain	20 d	R	Lumbar vertebra	+	DR + CT + MRI	L1 − L3	Y	Y	Y	10 yr	5
18	M/53	Han	Pain	1 yr	U	Lumbar vertebra		DR + CT + MRI	L5 − S1	Y	Y	Y	25 yr	2
19	M/40	Han	Pain	2 mo	R	Lung + thoracic vertebra + liver		DR + CT + MRI	L4 − S1		Y	Y	1 yr	1
20	F/25	Han	Pain	20 d	R	Lumbar vertebra	+	DR + CT + MRI	L5 − S3	Y	Y	Y	5 yr	3
21	M/56	Han	Pain	1 mo	R	Lung and sacrum	+	DR + CT + MRI	S1 − S4	Y	Y	Y	4 yr	1
22	F/54	Mongols	Pain	6 mo	U		−	DR + CT + MRI	S1 − S4		Y	Y	2 yr	0
23	M/62	Mongols	Pain	3 mo	R	Sacrum	−	DR + CT + MRI	S2 − S4	Y	Y	Y	9 yr	1
24	F/37	Han	pain	9 yr	R	Sacrum		DR + MRI	S1 − S3 + ilium	Y	Y	Y	2 yr	1
25	F/36	Kazakh	Pain	1 yr	R		−	DR + MRI	L5 − S3	Y	Y	Y	1 yr	0
26	F/56	Uygur	Pain	5 mo	R		+	CT + MRI	S1 − S3		Y	Y	1 yr	0
27	M/34	Han	Pain	3 mo	R	Liver	−	CT + MRI	Ilium	Y	Y	Y	1 yr	0
28	M/42	Uygur	Pain	3 yr	R		+	DR + CT + MRI	Ilium + ischium + pubis + hip		Y	Y	6 yr	1
29	F/38	Hui	Pain	3 yr	R	Thoracic vertebra	+	DR + CT + MRI	L4 − L5 + ilium + fumer		Y	Y	10 yr	1
30	M/27	Kazakh	Pain	2 mo	R		+	DR + CT + MRI	S1 − S5 + ilium	Y	Y	Y	5 yr	1
31	F/63	Hui	Pain	3 mo	U	Liver	+	DR + CT	Ilium		Y	Y	1 yr	0
32	F/55	Han	Pain	1 mo	R	Pelvis	+	DR + CT + MRI	Ilium		Y	Y	2 yr	1
33	F/19	Kazakh	Pain + limitation	4 yr	U	Live		DR + CT + MRI	Ilium + ischium + pubis + fumer	Y	Y	Y	1 yr	0
34	M/51	Kazakh	Pain	1 mo	R	Pelvis		DR + CT + MRI	Ilium	Y	Y	Y	3 yr	1
35	M/28	Uygur	Pain + lump	2 mo	R	Pelvis	+	DR + CT	Ilium		Y	Y	1 yr	0
36	F/22	Kazakh	Weakness	4 yr	R	Pelvis		DR + CT + MRI	S1 − S5 + ilium		Y	Y	1 yr	1
37	M/30	Kazakh	Pain	3 mo	R			DR + CT + MRI	Ilium + ischium + pubis + hip		Y	Y	2 yr	0
38	M/46	Uygur	Pain	4 mo	R		+	DR + CT	Ilium and hip		Y	Y	1 yr	0
39	M/49	Han	Pain + lump	1 yr	U	Liver		DR + CT	L4 − L5 + ilium + ischium + pubis	Y	Y	Y	2 yr	0
40	M/36	Han	Pain	9 yr	U	Pelvis		DR + CT	Ilium, ischium, and hip	Y	Y	Y	1 yr	1
41	F/31	Uygur	Pain	20 d	R	Pelvis	+	DR + CT	Ischium and hip	Y	Y	Y	1 yr	1
42	M/32	Kazakh	Pain	1 mo	R	Pelvis	+	DR + CT + MRI	Ilium		Y	Y	2 yr	1
43	F/39	Mongols	Lump	1 yr	R	Pelvis	−	CT	Ilium and hip	Y	Y	N	1 yr	0
44	M/44	Han	Pain	1 yr	R	Pelvis and liver	−	DR + CT + MRI	Ilium		Y	N	1 yr	4

*F*, false; *M*, male; *mo*, month; *d*, day; *yr*, year; *R*, rural; *U*, urban; *ELISA*, enzyme-linked immunosorbent assay; + , positive; − , negative; *DR*, digital radiology; *CT*, computed tomography; *MRI*, magnetic resonance imaging; *T*, thoracic vertebra; *L*, lumbar vertebra; *S*, sacral vertebra; *Y*, yes; *N*, no

The clinical manifestations varied significantly (Table 1). All patients presented different clinical symptoms depending on the site of infection, including pain (*n* = 41), weakness (*n* = 3), numbness (*n* = 2), progressive paraparesis (*n* = 2), and activity limitation (*n* = 1).. Before the patients were admitted to our hospital, the duration of the symptoms could range from 20 days to ten years. Most patients had mild to moderate disabilities, with sensory and autonomic symptoms predominating. Many patients can walk years after the onset of symptoms.

### Laboratory tests and pathological examination

All cases underwent pathological examination after the operation. Twenty-four patients were tested by ELISA, of which 18 (75%) were positive.

### Imaging findings

The location of echinococcosis of the vertebra and pelvis was summarized in Table 1. Of all patients, there are 24 cases of hydatid infection of the spine, 14 cases of hydatid infection of the pelvis and six cases of hydatid infection of both vertebra and pelvis. The site of infection were 13 (29.5%) thoracic, five (11.4%) lumbar, four (9.1%) lumbosacral, seven (15.9%) sacral, 19 (43.2%) ilium, seven (15.9%) hip, six (13.6%) ischium, five (11.4%) pubis, two (4.5%) femur, respectively. Seventeen cases were associated with intrahepatic or intrapulmonary echinococcosis, including nine liver involvement, seven lung involvement, four liver and lung involvement, and one pelvic involvement.

On X-ray examination, 25 (65.8%) cases revealed significant bone destruction, including eight (32.0%) cystic expansive destruction, 17 (68.0%) irregular osteolytic destruction. There were five (13.2%) irregular low-density shadows in the bone, and eight (21.1%) showed normal results. At the same time, there were the following accompanying manifestations: five (13.2%) calcification of hydatid sac, three (7.9%) soft tissue swelling, three (7.9%) vertebral space stenosis, three (7.9%) hip joint stenosis, three (7.9%) sacroiliac joint stenosis, and two (5.3%) round or oval mass in the surrounding soft tissue.

**Fig. 2:**
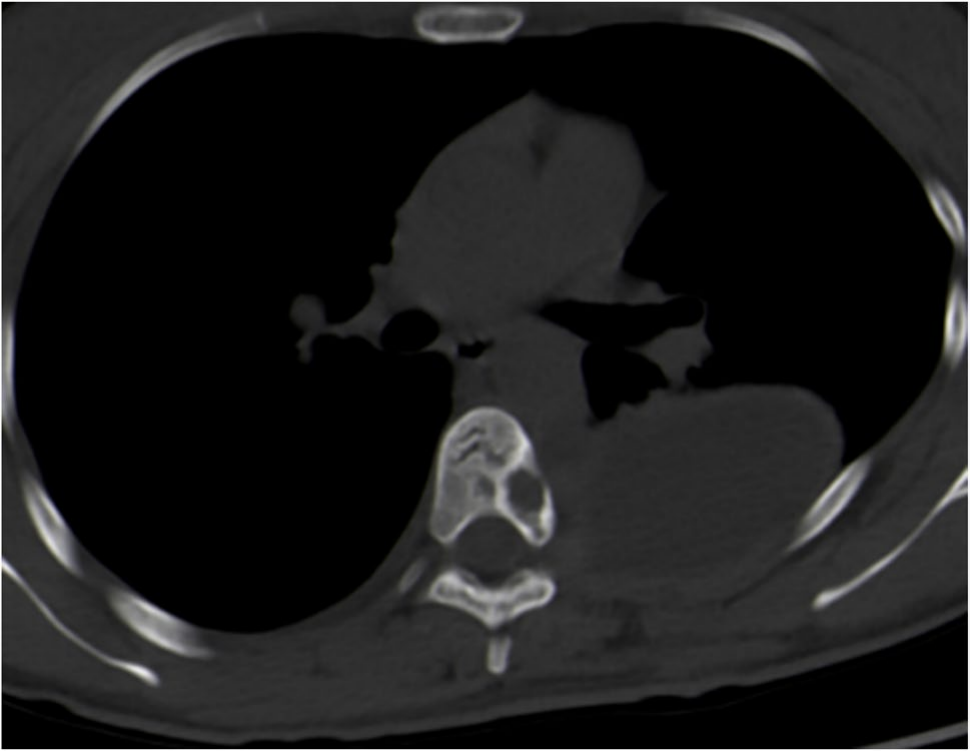
Plain CT scan showed cystic and expansive bone destruction of the 9th thoracic vertebrae with hydatid sac in the left soft tissue

**Fig. 3:**
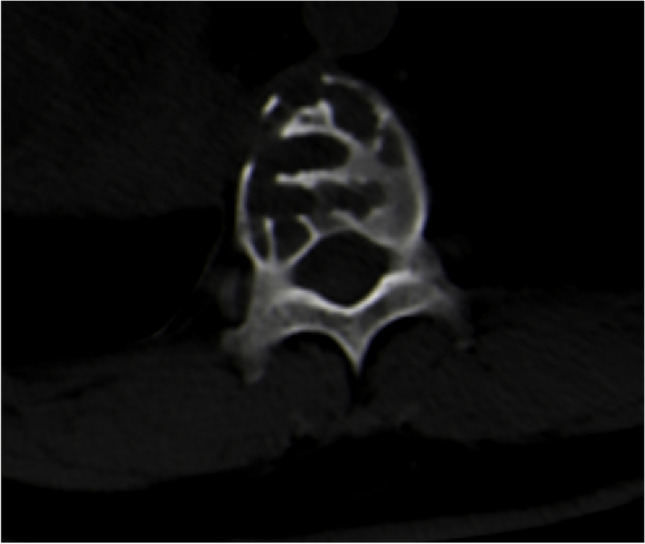
Plain CT scan showed different size bone destruction and dilated area with internal septum in the 11th thoracic vertebrae

**Fig. 4:**
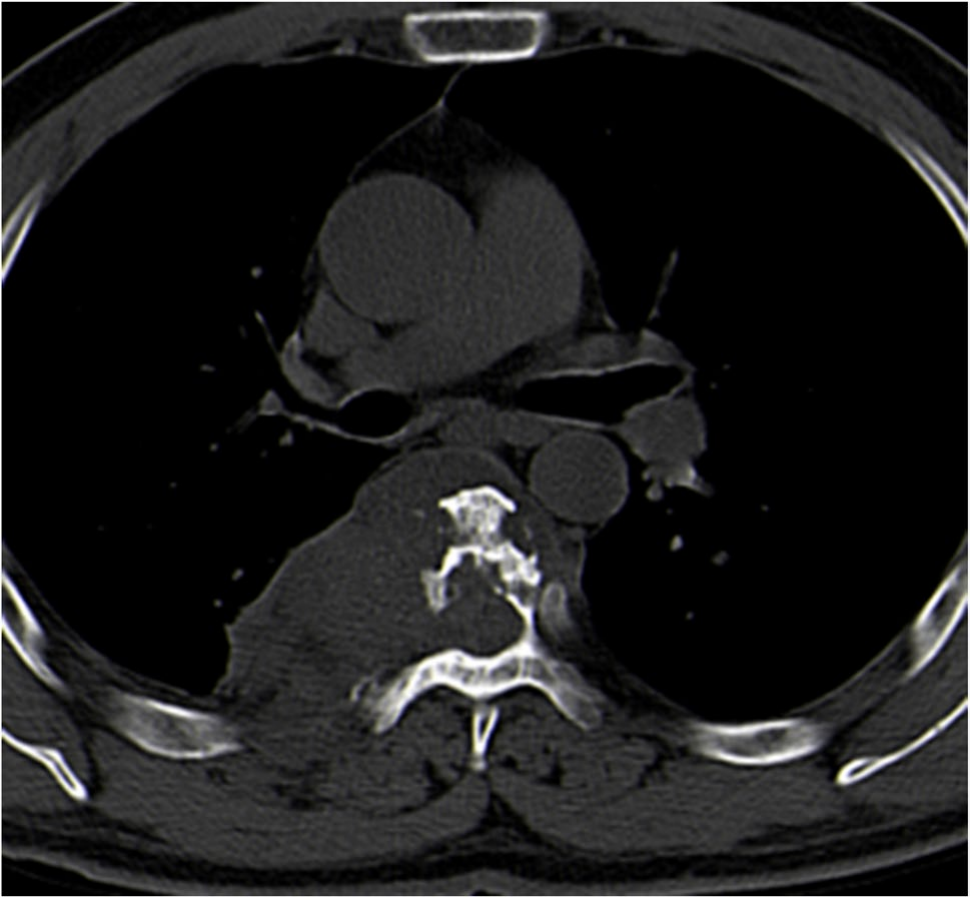
Plain CT scan revealed irregular bone destruction of the 7th thoracic vertebrae with hydatid cyst in surrounding soft tissue

**Fig. 5:**
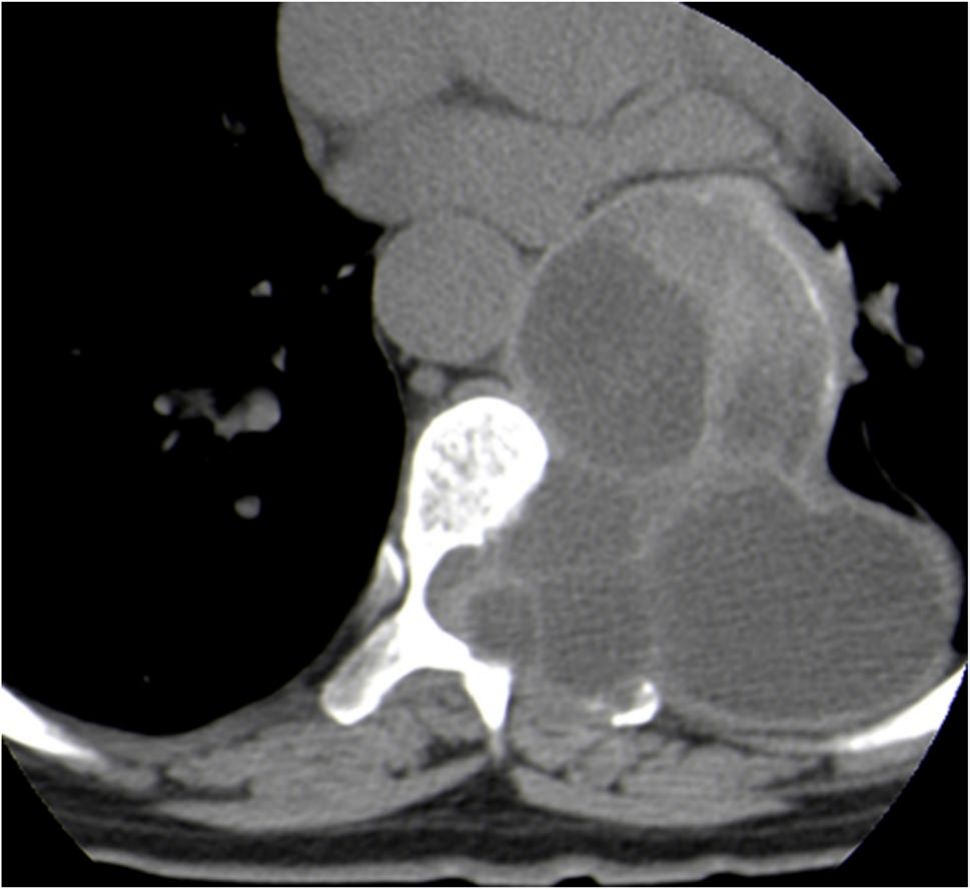
Coronal CT scan demonstrated cystic bone destruction in the first lumbar spine with a large hydatid cyst in the left soft tissue

**Fig. 6:**
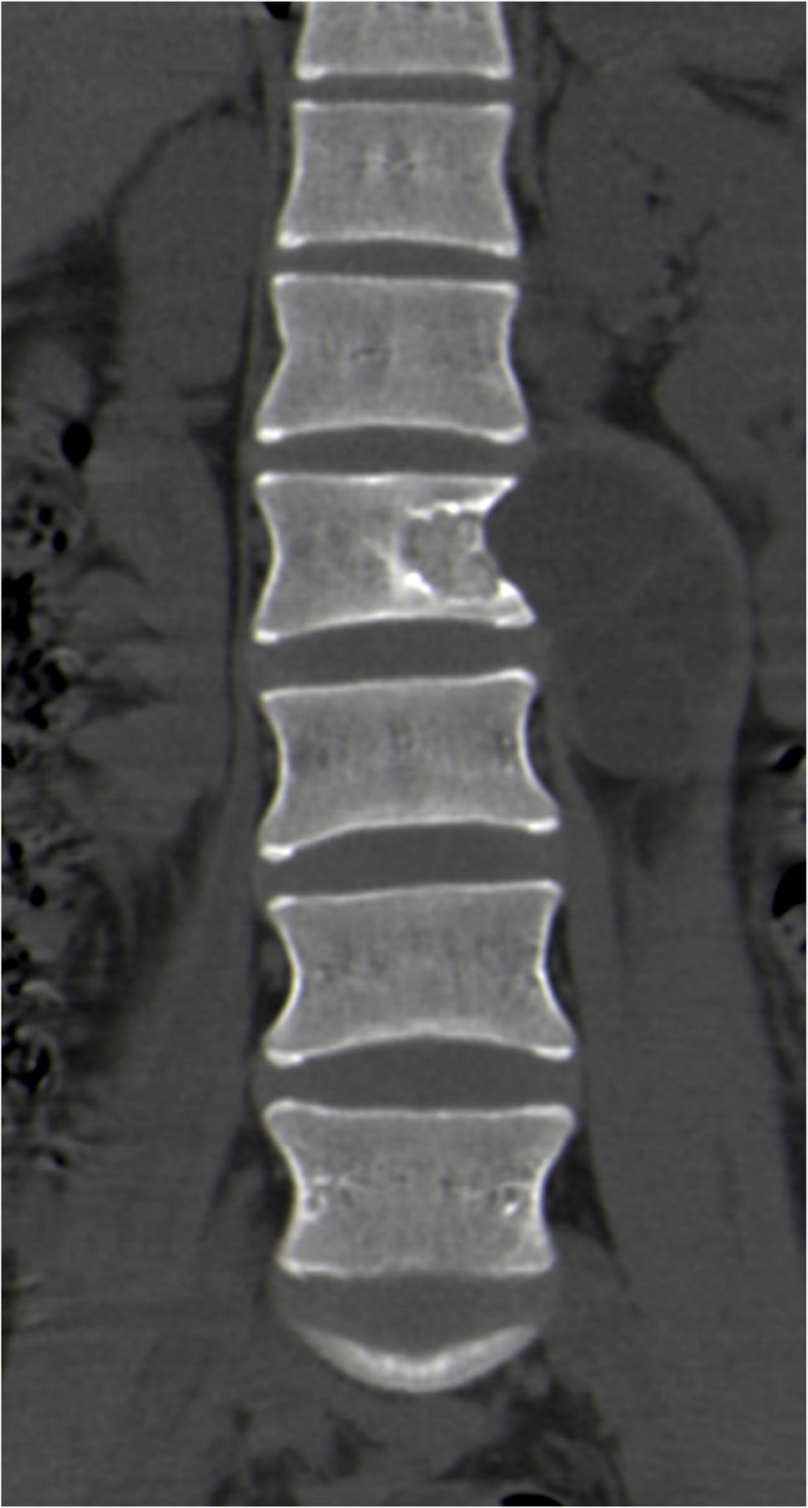
Axial CT scan revealed irregular bone destruction of the 10th thoracic vertebra and its left accessory with curved calcification of the cyst wall in a large hydatid cyst of the left soft tissue. The hydatid cyst entered the spinal canal and compressing the spinal cord

CT scan showed bone destruction in all cases, including 17 cystic dilated destruction (Fig. 2), eight different sizes of the dilated areas with the internal septum (Fig. 3), and 15 irregular destructions (Fig. 4). Three cases had a secondary pathological fractures. There were 21 vertebral attachment invasions and eight ribs invasions. There were 11 compressions of the spinal canal and 22 invasions of the surrounding soft tissues (paraspinal muscles, iliac muscles, iliopsoas muscles, gluteus muscles, spinous muscles, internal obturator muscles, etc.) (Fig. 5). There were 13 single cystic types, nine polycystic types, and 18 irregular types. The cyst is a round, watery, well-defined shadow, while the daughter cyst in the polycystic mother cyst is a round, low-density shadow in the septum. There were 14 calcification in the cyst or cyst wall (Fig. 6), and two lymph node enlargements. The intervertebral space was narrowed in 11 cases, and the sacroiliac joint was narrowed in seven patients. Of the nine cases of hydatid disease, seven cases showed no enhancement.

**Fig. 7:**
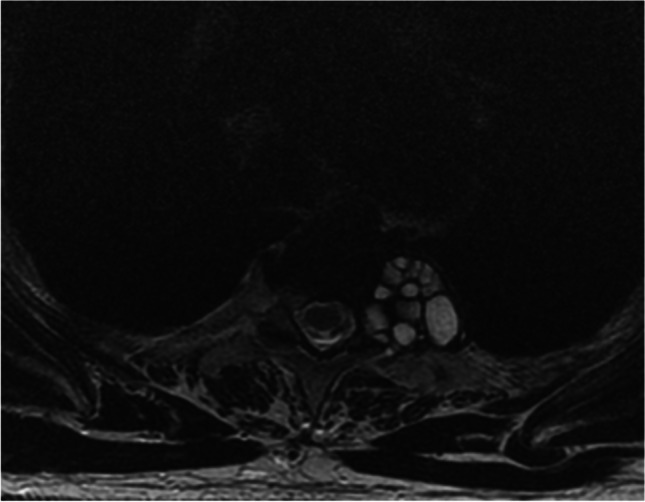
Axial MRT2 image showed the typical multilobular, multicystic septated mass lesions on the left side of the 9th thoracic vertebra

**Fig. 8:**
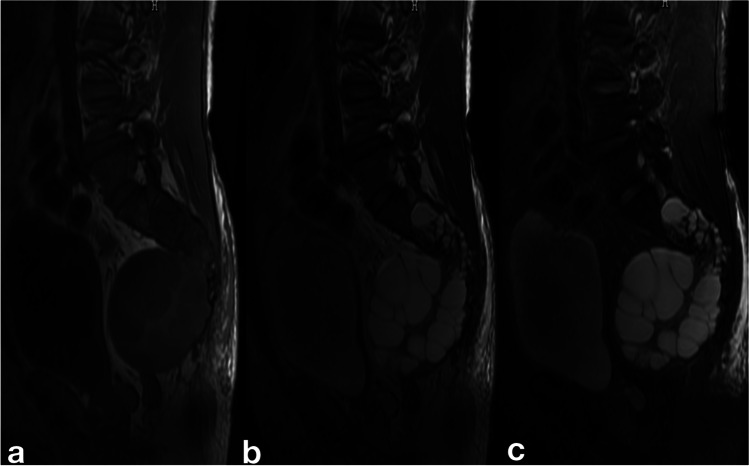
a–c Sagittal MR imaging demonstrated a polycystic round lesion with low signal intensity on T1WI (**a**), high signal intensity on T2WI (**b**), and high signal intensity on STIR (**c**). There was linear low signal on T1WI, T2WI, and STIR in the septa and cyst’s wall. The damaged vertebrae ranged from the 2nd to the 5th sacral vertebra

Of 35 cases, MRI findings showed bone destruction in all cases. The cystic dilated destruction in twenty-two cases showed low signal intensity on T1WI and high signal intensity on T2WI. Among them, 20 cases showed intracystic septal; The septal and cyst wall were low linear signal on T1WI, T2WI, and Short-tau inversion recovery (STIR) sequences (Fig. 7). The well-defined peripheral soft tissue cystic lesions in fourteen patients were low signal on T1WI, high signal on T2WI, and high signal on STIR (Fig. 8a–c). On contrast-enhanced T1-weighted images of 11 patients, the cystic lesions showed no enhancement in seven cases. Still, mild linear enhancement at the edge of the lesion and partial interval enhancement was observed in four cases.

### Treatment and follow-up

The surgical therapy, medical therapy, and follow-up time were summarized in Table 1. All patients were treated with albendazole before and after surgery. All patients were followed up for at least one year with radiological examination for evaluation. The mean follow-up time was 3.6 (1-25 years) years.

## Discussion

Hydatidosis is a severe economic and public health problem in regions where hydatid disease is endemic. In epidemic areas, the annual incidence of CE ranges from < 1 to 200 per 100,000 [6]. CE mortality is low, ranging from 2 to 4%, but may increase significantly if not adequately managed. The current estimated global burden of CE averages 285,500 disability-adjusted life years (DALYs) [7, 8]. The World Health Organization(WHO) has listed echinococcosis as one of the 17 neglected diseases targeted forcontrol or elimination by 2050. Human bone echinococcosis is extremely rare, even in areas where hydatidosis is endemic [9–13]. Due to the rarity of hydatidosis and the lack of specificity of early clinical symptoms, it often leads to misdiagnosis and missed diagnosis. This disease is difficult to eradicate, especially when it involves the spine. Due to the complex structure of the vertebral body and its appendages, the disease is difficult to be completely removed, so the recurrence rate and disability rate are high, which not only brings great suffering and economic burden to the patients' families, but also seriously affects the survival and quality of life of the patients. According to a study in Iran, the cost of care and treatment for this disease exceeds 232.3 million US$ per year [14].China's Xinjiang is one of the provinces most affected by hydatid disease. In the current study, there was the most significant number of cases with bone echinococcosis in the world.

Bone echinococcosis is a rare disease with a low incidence (0.5–4.0%) [12, 13]. Vertebrae is the most commonly infected site (50%), followed by the pelvis (25%) and long bone (15–25%) [11, 15]. Bone echinococcosis generally grows slowly, ranging from a few months to many years. Most infections occur in childhood and are not diagnosed until adulthood [2]. In our study, all patients are adults and the mean age was 43 years (25 males, 19 females; age range 19–68 years). Thirty-two patients came from rural areas and 12 patients came from urban. Four patients had risk factors for close contact with dogs, cattle or sheep.

Bone echinococcosis usually takes ten to 20 years for the clinical manifestation to become obvious, and it is usually detected after secondary infection or compression of adjacent soft tissues or nerves [16, 17]. The clinical presentation usually depends on the size of the cyst and the organ system involved. Early cysts are small and usually asymptomatic. With the progress of the disease and the enlargement of the cyst, the patient began to suffer from continuous pain, which gradually led to severe neurological impairment and different degrees of limb weakness, and even paralysis [18–20]. Due to the lack of characteristic signs and symptoms, spinal CE may present as any symptom related to vertebral bone destruction or spinal cord compression. The most common are long-term back pain and / or subacute symptoms associated with spinal cord or spinal nerve compression (radicular pain, peripheral sensitivity loss, sphincter disturbance, bladder dysfunction, paraparesis, paraplegia) [11]. Our results showed that the similar clinical manifestations: pain (*n* = 41), weakness (*n* = 3), numbness (*n* = 2), progressive paralysis (*n* = 2), and activity limitation (*n* = 1). In the current study, pain included thoracic back pain, back pain, lumbar back pain, hip pain, lower back pain, sacral tail pain, and lower limb pain. In the published literature, sciatica caused by compression of lumbosacral nerves has also been described as the first clinical symptom of pelvic echinococcosis [21].

The serological test is a valuable diagnostic investigation for hydatid disease, but it sometimes showed false positive and false negative results, which is why the serological cannot serve as the most appropriate early diagnostic tool for mass screening in in areas of endemicity [16]. Therefore, serology is now commonly used to confirm imaging results; it can also be for a specific geographic area and specific people infected with pressure to provide some insights [7]. Antigen 5 is widely used in serological assays for CE [22]. In our study, 24 patients underwent ELISA, of which 18 (75%) were positive. However, echinococcosis of bone is often associated with echinococcosis of other sites. Thus, bone hydatid disease can be missed, and the diagnosis requires other tests. Pathologic examination of the bony lesion after surgical excision is the gold standard for definitive diagnosis. Pathological examination was performed in all cases after the operation.

Radiological examination plays an essential role in the diagnosis of bone hydatid. Traditional X-ray plain is preferred to detect bone hydatid disease, which is characterized by cystic or irregular bone destruction. The calcification can sometimes be seen in the cyst wall or intracyst. In the spine and pelvic, the main manifestation is irregular bone destruction, sometimes invasion of vertebral accessory or adjacent soft tissue, and sometimes the involved intervertebral space or joint may narrow or disappear. The X-ray appearance of bone echinococcosis is nonspecific. In the current study, 25 cases showed cystic or irregular osteolytic bone destruction, no periosteal reaction, and surrounding with or without calcification. The involved intervertebral space or joint space is generally unchanged and narrow. The X-ray appearance of bone echinococcosis that is nonspecific is influenced mainly by the location of the cyst and associated complications, such as secondary infection and rupture, so definite diagnosis sometimes needs to be combined with clinical history and further imaging examination.

CT findings of osteohydatidosis showed typical osteolysis with clear boundaries, sometimes with coarse trabeculae within it, forming a honeycomb appearance, accompanied by an expansion of the bone and thinning of the bone cortex [23]. It has been suggested in the published literature that the round or ovoid space-occupying lesion with “double layer arcuate calcification” is a characteristic feature of hydatid cysts caused by bone echinococcus infection that is distinct from other cystic diseases [24]. There is generally no periosteum reaction in bone echinococcosis. When the lesion breaks through the bone cortex and invades the surrounding soft tissue, it presents as a round or ovoid cystic mass with a sharp, thin margin, and no contrast enhancement. The cyst wall showed linear enhancement. The CT and its three-dimensional reconstruction technology can accurately evaluate the bone destruction, calcification, and pathological fracture of osteohydatidosis and provide more information for the anatomical location of the lesions [25]. In this study, there were 32.5% single cystic type, 22.5% polycystic type, and 45% irregular type; the cyst is a round, watery, and well-defined shadow, while the daughter cyst is in the polycystic mother cyst that is a round, low-density shadow in the septum. There was 35% calcification in the cyst or cyst wall; the intervertebral space was narrowed in 11 cases, and the sacroiliac joint was narrowed in seven cases. Therefore, CT technique can be used as a common diagnostic method of osteohydatidosis.

MRI technique is a better alternative because it can show precise anatomical localization and extension of bone echinococcosis. It can observe the morphological characteristics, scope and location from the sagittal, coronal, and axial planes and has a special value in showing the relationship between the surrounding tissues and organs of cystic bone echinococcosis. A single echinococcosis cyst involving bone and surrounding soft tissue showed low or intermediate signal intensity on T1-weighted images and hyperintensity on T2-weighted images. In the current study, the cystic-dilated destruction in 62.9% cases showed low signal intensity on T1WI and high signal intensity on T2WI; the well-defined peripheral soft tissue cystic lesions in 40% of cases were low signal on T1WI, high signal on T2WI, and high signal on STIR; on contrast-enhanced T1-weighted images of 11 patients, the cystic lesions showed no enhancement in seven cases, but mild linear enhancement at the edge of the lesion and partial interval enhancement was observed in four cases. However, when a hydatid cyst ruptured or infected, the signal of T1WI and T2WI is significantly enhanced, especially on T2WI, and the boundary of the cyst changes from sharp to blurred. Relevant published literature also proves this point [25, 26]. The typical characteristic of polycystic type on MRI is the appearance of multiple cysts (daughter cysts) with different signal intensity within the larger cyst, forming “small vesicle” high daughter cysts. The signal from the daughter cysts was low in relation to the fluid in the cyst on T1-weighted images and high on T2-weighted images. MRI is the most useful tool for the diagnosis of hydatid cysts [24].

Surgery is the most common treatment for bone hydatidosis. Chemotherapy can be employed as neoadjuvant therapy to shrink the cyst load before surgery or as adjuvant therapy to decrease the recurrence risk [2]. In the current study, 42 patients underwent surgery after diagnosing bone hydatids, and all were treated with albendazole before and after the operation. However, residual parasite material due to partial excision has a high potential to reactivate parasite growth in the future [13]. But in many locations, such as the spine, pelvis, and hip, radical surgery is nearly impossible, the results are disappointing even after aggressive treatments, and they often show frequent recurrences [27, 28]. In the current study, All patients were followed up for an average of 3.6 years (range from 1 to 25 years), and 23 (52.2%) patients relapsed. Sixteen patients had two operations, four patients of three operations, one patient of four operations, and one patient of five operations. The operative area was filled with bone cement in three patients. Bone cement filling is one of the effective methods to reduce the recurrence rate after surgical resection [2, 18, 29, 30].

Differential diagnosis includes bone tuberculosis, mycoses, benign cystic lesion of bone, metastatic disease, and other neoplastic lesions [25, 31]. Epidemiological evidence, radiological examinations combined with laboratory analysis of infectious markers, and positive echinococcosis serology can help doctors diagnose bone echinococcosis earlier and correctly.

There are limitations to this study. Bone echinococcosis is rare, and there were too few cases in this study. This was a retrospective study on the clinical signs, laboratory and imaging manifestations, and treatment. A follow-up study is underway.

In conclusion, even in epidemic areas, the incidence of bone echinococcosis is relatively rare. However, when encountering the vertebral and pelvic destruction, consider bone echinococcosis’s possibility, especially for the herdsmen in endemic regions.

## Data Availability

The data sets cannot be made publicly available, and restrictions apply to the availability of these data. The data can be requested from the authors and require permission from the Xinjiang Medical University Affiliated First Hospital.

## Abbreviations

ELISA: Enzyme-linked immunosorbent assay
CT: Computed tomography
MRI: Magnetic resonance imaging
STIR: Short-tau inversion recovery
